# Corrigendum to “Genetic Diversity in Drug Transporters: Impact in African Populations”

**DOI:** 10.1111/cts.13156

**Published:** 2021-10-20

The article by Rajman et al. (2020),^1^ was published with an error in Table 1. In the first row of the column “Expression site,” intestine, kidney, and brain should be removed.
